# Pesticides in Honeybee Products—Determination of Pesticides in Bee Pollen, Propolis, and Royal Jelly from Polish Apiary

**DOI:** 10.3390/molecules30020275

**Published:** 2025-01-12

**Authors:** Agata Swiatly-Blaszkiewicz, Agnieszka Klupczynska-Gabryszak, Eliza Matuszewska-Mach, Joanna Matysiak, Everaldo Attard, Dariusz Kowalczyk, Aleksandra Adamkiewicz, Bogumiła Kupcewicz, Jan Matysiak

**Affiliations:** 1 Department of Inorganic and Analytical Chemistry, Faculty of Pharmacy, Collegium Medicum in Bydgoszcz, Nicolaus Copernicus University in Toruń, Jurasza 2, 85-089 Bydgoszcz, Poland; kupcewicz@cm.umk.pl; 2 Department of Inorganic and Analytical Chemistry, Poznan University of Medical Sciences, 60-780 Poznan, Poland; aklupczynska@ump.edu.pl (A.K.-G.); eliza.matuszewska@ump.edu.pl (E.M.-M.); jmatysiak@ump.edu.pl (J.M.); 3 Faculty of Health Sciences, University of Kalisz, 62-800 Kalisz, Poland; jkamatysiak@gmail.com (J.M.); d.kowalczyk@uniwersytetkaliski.edu.pl (D.K.); olamusiol92@gmail.com (A.A.); 4 Division of Rural Sciences and Food Systems, Institute of Earth Systems, University of Malta, MSD2080 Msida, Malta; everaldo.attard@um.edu.mt

**Keywords:** pesticides, *Apis mellifera*, pollen, royal jelly, propolis

## Abstract

The bioaccumulation of pesticides in honeybee products (HBPs) should be studied for a number of reasons. The presence of pesticides in HBPs can provide new data on the risk related to the use of pesticides and their role in bee colony losses. Moreover, the degree of contamination of HBPs can lower their quality, weaken their beneficial properties, and, in consequence, may endanger human health. The aim of this study was to quantify a broad range of pesticide residues in three different HBPs—bee pollen, propolis, and royal jelly. Samples were collected in the years 2017–2019 from the apiary in west-central Poland. Bee products were analyzed for the presence of over 550 pesticides using the QuEChERS (Quick, Easy, Cheap, Effective, Rugged, and Safe) method. Twenty-nine of the contaminants were quantified at least in one of the samples. Nine of them exceeded the maximum residue levels for honey. It should be noted that any dose of pesticides can cause a health hazard due to toxicity, since these substances may act synergistically. This current study revealed the high need for the pesticide monitoring of HBPs and proved that there is a need to expand the European Union Pesticides Database to include more HBPs.

## 1. Introduction

Honeybees (Apis mellifera) play a crucial role in agriculture and floral ecosystems. However, recently, there was a significant decline in the number of pollinators (including bees). Since almost 90% of the world’s flowering plant species depend on pollinator insects or other animals, this decline has become a major environmental issue [1]. A specific syndrome which is responsible for bee colony losses is called colony collapse disorder (CCD). In CCD, forager bees disappear because they cannot return to the hive. Therefore, adult bees abandon their colony, leaving immature bees without food and nourishment. There are several factors that are responsible for CCD: diseases, infections with mites and pathogen microorganisms, pesticides, and deforestation [2]. It should be noted that in many papers, pesticides and environmental pollution are pointed out as the main causes of CCD [3,4]. Due to the many important roles of honeybees in the environment and agriculture, their health has become a matter of public concern.

The use of pesticides, including insecticides, herbicides, or fungicides, has a great impact on increasing food production or improving food quality. On the other hand, the negative effect on pollinators may lead to the collapse of many bee colonies [5]. Compared to other insects, bees are extremely sensitive to chemicals because they do not have a sufficient number of detoxifying enzymes [6]. Even at very low concentration levels, pesticides can be harmful to bees. Sub-lethal doses of insecticides influence the cognitive abilities of bees and weaken their immune systems, and consequently open the way to parasite and viral infections. Moreover, insecticides impair their orientation, make them weak and unable to fly [7].

Obviously, pesticides can negatively impact bee colonies. However, pesticide contamination of honeybee products (HBPs) may also influence food safety. HBPs, including pollen, royal jelly, and propolis, are widely used for food supplementation. Therefore, the bioaccumulation of pesticides should be constantly monitored. A Regulation at European Union-level (No 396/2005) was devised in order to define maximum residue levels (MRLs) of active substances present in food and feedstuffs. Within the European Union Pesticides Database, MRLs for honey can be found, and there are no defined values for other HBPs [8].

The bioaccumulation of pesticides in various HBPs should be studied for several reasons. The presence of pesticides in HBPs can provide new data on the risk related to the use of pesticides and their role in bee colony losses. The degree of contamination of HBPs can lower their quality, weaken their beneficial properties, and, in consequence, put human health at risk [9]. Moreover, research conducted on HBPs may be used in the biomonitoring of the foraging area in regard to environmental contaminants [10].

The analysis of pesticide contaminations is an increasingly challenging aspect. It should be noted that pesticides are ubiquitous pollutants found in surface and groundwater, similar to antibiotics and drugs [11]. Therefore, it is necessary to apply a sophisticated methodology for quantifying contaminants in different matrices. The main difficulties in determining pollutants include their instability and/or precipitation, degradation by-products, and also properties of a chemical reaction [12]. The importance of studies focusing on the stability of contaminants and the validation of the quantitative method should be emphasized.

Most of the studies on HBPs focused only on the determination of pesticides levels in bee pollen and honey [8,13,14,15]. Despite plenty of HBPs, scarce information on pesticide contamination can be found in the literature. Therefore, we also included in our research other important HBPs and we decided to examine bee pollen, royal jelly, and propolis. They are essential for bees and have also become very popular as food supplements for humans. The aim of our study was to quantify over 550 pesticide residues in three different HBPs. The studied samples were collected between 2017 and 2019 from the apiary in west-central Poland. In order to provide reliable results, two sophisticated analytical methods were used: gas and liquid chromatography coupled with tandem mass spectrometry (GC-MS/MS and LC-MS/MS). This is the first study that compares the content of pesticide residues in such a set of HBPs. The obtained results may contribute to the valuable assessment of pesticide health risks to the honeybees and food safety implications. Moreover, the interesting sampling area makes the present study a significant contribution to the knowledge on HBP pesticide contamination in Europe.

## 2. Results

The applied methodology allowed us to test for over 550 pesticide residues in HBPs: pollen, propolis, and royal jelly. Twenty-nine of the studied substances exceeded the LOQ at least in one of the samples. Table 1 and Table 2 present the mean concentration values, and the RSD of all measurements did not exceed 10%. Table 1 shows the results of the GC-MS/MS pesticide screen, and Table 2 shows the LC-MS/MS pesticide screen findings. In the studied samples, we identified 9 insecticides, 15 fungicides, and 5 herbicides. The presence and concentration of the substances varied depending on different points of time and the type of bee product. Table 3 contains information about 9 pesticides, which had concentrations higher than MRLs for honey at least in one of the samples.

The most prevalent pesticide determined in the studied HBPs was chlorpyrifos. It was present in almost every pollen and propolis sample (except pollen collected in July 2019), with a concentration range between 0.014 mg/kg and 0.4 mg/kg. Azoxystrobin was found in five pollen samples, and in two of them, the concentrations were above MRLs approved for honey—August 2017 (2.9 mg/kg) and May 2018 (0.57 mg/kg). Three pollen samples were contaminated with fenvalerate, and in one of them, the concentration exceeded MRLs allowed for honey (August 2017 with concentration of 0.058 mg/kg). Boscalid, cypermethrin, cyprodinil, tebuconazole, and propamocarb hydrochloride occurred in single pollen samples. The most contaminated sample was pollen collected in Aug 2017 and it contained boscalid (0.15 mg/kg), chlorpyrifos (0.4 mg/kg), cypermethrin (0.08 mg/kg), fenvalerate (0.058 mg/kg), and azoxystrobin (2.9 mg/kg). Concentrations of all of these pesticides were above the MRLs approved for honey.

Interestingly, both propolis samples, collected in 2018 and 2019, were significantly contaminated with chlorpyrifos and bifenyl. Except for one pollen sample (May 2019), bifenyl occurred only in propolis samples. Therefore, it was not even found in the corresponding pollen samples collected in the same point of time. The concentrations of bifenyl were very similar in both propolis samples: in the sample collected in June 2018, it was 0.039 mg/kg, and in the sample collected in June 2019, it was 0.042 mg/kg. The royal jelly sample collected in June 2019 contained all assayed pesticide residues below the reporting limit (<0.01 mg/kg).

## 3. Discussion

The research provided novel data on the pesticide residue content in three HBPs: bee pollen, propolis, and royal jelly collected in west-central Poland. Our study revealed the presence of various pesticides in two types of HBPs: bee pollen and propolis. Interestingly, the royal jelly was not contaminated with any of the examined chemicals. Each pollen and propolis sample demonstrated its unique contamination profile. The results may suggest that honeybees were exposed to multiple pesticides, which can affect their health. The obtained results showed that the pesticide screening and monitoring of HBPs are fully justified.

The presented methodology allowed us to the analyze over 550 pesticides in the bee product samples. HBPs are complex matrices, resulting in many challenges related to their analysis. Therefore, the analysis of contaminants in bee-related samples requires selective and sensitive instrumentation capable of detecting hundreds of compounds in a single run, such as liquid chromatography/gas chromatography coupled to tandem mass spectrometry. One of the major steps of the analysis is the proper extraction method, which enables the efficient isolation of pesticides and influences the final results. In the literature, different techniques were used in the pesticide area: solid-phase extraction (SPE) [16], liquid–liquid extraction (LLE) [17], matrix solid-phase dispersion (MSPD) [18], and QuEChERS (Quick, Easy, Cheap, Effective, Rugged, and Safe) [19,20,21,22]. Recently, QuEChERS has become the most popular extraction and clean-up method for pesticide quantification. This technique is characterized by a simple and fast procedure and the possibility of facilitating the detection of many compounds simultaneously. Therefore, the consumption of the chemical reagents is lower compared to other techniques, which is in agreement with the Green Chemistry policy [23]. QuEChERS has progressively replaced other laborious, time-consuming, and non-environmental-friendly extraction methods. R. Perestrelo et al. summarized the comparison of the efficiency of different extraction approaches for the analysis of pesticides [24]. Generally, QuEChERS has a number of advantages: time-saving, high sample throughput, less labour, and wide analytical scope. Moreover, it was mainly proved to be most efficient method among others. The QuEChERS method has better efficiency than SPE and LLE for the determination of pesticides in roots and rhizomes of Chinese herbal medicines [25], as well as in honey and honeybees [26]. However, in some specific applications, this trend has not been confirmed. For example, SPE showed better performance than QuEChERS in the clean-up for the analysis of mycotoxins in fruits and vegetables. SPE was presented as a more selective, accurate, and precise method in mycotoxin determination [27]. In the present study, a wide range of pesticides were analyzed. Therefore, QuEChERS was used as a method of choice for the analysis of multiclass pesticide residues in various type of samples.

The variation in pesticide content across different bee products is likely due to the different ways in which the bees produce them [28]. Bee pollen is formed by forager bees which gather pollen from a variety of flowers and then combine it with nectar and secretions from their salivary glands to create small pellets [29]. Propolis, a resinous substance, is produced by bees through the combination of plant secretions and pollen with their saliva and beeswax [30]. Royal jelly, a milky secretion from worker bees’ hypopharyngeal glands, stands apart from bee pollen and propolis as it contains no plant material [31]. Bee pollen and propolis consist mainly of plant parts and secretions, which may absorb chemicals, including plant protection products. Thus, these honeybee products reflect the environmental contamination with pesticides (and also other pollutants, such as heavy metals [28]). In contrast, royal jelly, being a pure secretion of the bees’ throat glands, contains no detectable levels of pesticides. The absence of pesticides in royal jelly suggests that, although pesticides may accumulate in bees’ bodies [19,32], the amount excreted through royal jelly is negligible.

Our study confirmed the presence of pesticide residues in bee pollen and propolis samples and provided qualitative and quantitative data on pesticide residues present in HBPs of Polish origin. However, royal jelly was free from any contamination. This result is in agreement with other papers [33,34,35]. The experiment conducted by Böhme et al. [35] was designed to imitate an environment with high pesticide contamination. Adult honeybees were fed with special pollen containing a cocktail of 13 toxic substances. However, their concentration in royal jelly was still extraordinary low [35]. Therefore, it can be suspected that pesticide residues in pollen do not affect the development of queens [36]. The absence of pesticide residues in the royal jelly samples is fully justified and does not indicate any limitations of the methodology used. Detailed information on royal jelly contamination and proper sample preparation was included in the review paper [34].

The studied HBPs, especially bee pollen, contained different pesticide classes (fungicides, insecticides, and herbicides) and groups (neonicotinoids, organophosphates, pyrethroids, triazoles). The most important group of neurotoxins is the neonicotinoid group, which includes imidacloprid, acetamiprid, clothianidin, thiamethoxam, thiacloprid, dinotefuran, imidalothiz, and nitenpyram, amongst others [19]. Neonicotinoids are antagonists of insect acetylcholine receptors and they have been widely used since 1991 [37]. Thiacloprid and acetamiprid were found in the studied samples, which are less toxic to bees compared to other pesticides from this group. Therefore, they are often applied to flowering crops. Their concentrations were below the MRLs approved for honey. However, their presence might still affect bees’ health. Even sub-lethal concentrations may have an impact on bee neuronal functions, orientation, and survival under starvation and pathological stress [38,39,40,41]. Moreover, samples that contained neonicotinoids (thiacloprid and/or acetamiprid) also contained high concentrations of some fungicides. According to the literature, neonicotinoids and fungicides interact synergistically, and the toxic side-effects on bees may be amplified [42,43].

Another group of highly toxic insecticides is organophosphate pesticides. They inhibit the activity of cholinesterase in the body of bees. As a result, cholinergic synapses are over-stimulated, leading to excitatory paralysis and death [44]. Chlorpyrifos, as a member of this group of pesticides, was identified in almost all of the studied samples (87.7% of pollen samples). However, according to its potential neurotoxicity and reproductive toxicity for humans, it has been prohibited in the European Union since 2020 [43].

High concentrations of cypermethrin and fenvalerate were also detected in one of the pollen samples (Table 3). These insecticides belong to the pyrethroid group of pesticides. They are widely used because of their low toxicity to humans. However, pyrethroids can block the voltage-gated sodium channels, which leads to convulsion and death of the bees and other insects [45,46].

The majority of studies focus on bees’ exposure to insecticides. Nevertheless, non-insecticidal chemicals including fungicides may also have severe sub-lethal and lethal effects on bees [42]. It should be noted that in the presented study, as many as six fungicides exceeded the MRL permitted for honey (biphenyl, boscalid, cyprodinil, tebuconazole, azocystrobin, propamocarb). Triazoles (boscalid and tebuconazole), as well as strobilurin fungicides (azoxystrobin), are one the main classes of pesticides used worldwide [47,48]. Generally, they are characterized by low toxicity to birds, mammals, and insects. However, as with other potential toxins, the most important factors are the duration and degree of total exposure to pesticides. Moreover, many papers emphasized that the presence of fungicides may strongly increase the toxicity of insecticides like neonicotinoids and pyrethroids [49].

Due to the presence of pesticides in HBPs, HBPs can be used as potential bioindicators of environmental pesticide contamination. Therefore, a number of studies were performed to monitor pesticide residues in HBPs in different areas including Spain [50,51], France [52], Switzerland [10], Poland [53], Germany [54], Brazil [55,56], China [56], and Taiwan [57]. Pollen samples collected in Spain were mostly contaminated with miticides, chlorpyrifos (0.001–0.100 mg/kg), and acetamiprid (0.007–0.104 mg/kg) [50]. HBPs from France (bee bread and beeswax) contained predominantly neonicotinoids (including acetamiprid, imidacloprid, thiacloprid) and boscalid [52]. Our results are also consistent with the Swiss study, where several common pesticides were detected in HBPs (bee bread): acetamiprid (max. 0.016 mg/kg), thiacloprid (max. 0.037 mg/kg), azoxystrobin (max. 0.072 mg/kg), boscalid (max. 0.050 mg/kg), cyprodinil (max. 1.965 mg/kg), difeconazole (max. 0.073 mg/kg), prosulfocarb (max. 0.038 mg/kg), and terbuthylazine (max. 0.026 mg/kg) [10]. The study conducted in Poland in 2016 [53] identified 29 pesticide residues in bee pollen samples (13 of them are common with our research). Interestingly, compounds most frequently found included tebuconazole (0.004–0.064 mg/kg), thiacloprid (0.061–0.136 mg/kg), and chlorpyrifos (0.015–0.040 mg/kg), which were also detected in our study.

It should be noted that there are only few papers focusing on pesticide residues in propolis from Europe [51,54]. Furthermore, there is no research on pesticides present in propolis of Polish origin. The absence of pesticides was reported in the propolis samples derived from Spain [51] and Germany [54]. The qualitative HBP results obtained in our study are consistent with other publications from Europe. However, it can be noticed that most of the detected contaminants occur in higher concentration levels than in other parts of Europe. Our finding is in agreement with [58], which showed that the consumption of pesticides in Poland is among the highest in Europe. Different pesticide residues were found in bee pollen from South America (Brazil) and Asia (Taiwan). Research conducted in Brazil revealed the presence of bioallethrin (pyrethroid) and pendimethalin (dinitroaniline herbicide) in pollen samples [55]. On the other hand, HBPs (pollen) from Taiwan contained fluvalinate, chlorpyrifos, carbendazim, carbaryl, chlorfenapyr, imidacloprid, ethion, and flufenoxuron [57]. Propolis samples from Brazil (South America) and China (Asia) were mostly contaminated with fluvalinate (0.012–0.587 mg/kg) [56]. Fluvalinate was also detected in propolis samples in our study; however, the concentration level was smaller (0.05 mg/kg). Candies containing propolis from the Mercosur region (South America) were contaminated with coumaphos (0.004–0.027 mg/kg) and chlorpyrifos (0.010–0.021 mg/kg) residues [59]. Bearing in mind that candies are processed food, it is not possible to compare the level of pesticide residues with the raw propolis material.

## 4. Materials and Methods

### 4.1. Sample Collection

Samples of bee pollen, propolis, and royal jelly were directly harvested from Apis mellifera hives located in the village of Góry Złotnickie (coordinates: N 51°87′504″, E 18°12′431″) within the Greater Poland Voivodeship, west-central Poland. The collection of bee pollen occurred during the summer season between August 2018 and July 2019. Propolis samples were collected in June 2018 and June 2019. Additionally, a pilot study was conducted using a royal jelly sample collected in 2019. All samples were stored in darkness at −80 °C until analysis.

### 4.2. The Determination of Pesticides

The studied samples were prepared using QuEChers extraction methods [58]. Firstly, the samples were homogenized. Then, they were extracted using acetonitrile, magnesium sulphate, and citrate salts. The mixtures were vigorously shaken and then centrifuged to achieve phase separation. The organic fractions underwent clean-up via dispersive solid-phase extraction (D-SPE), utilizing bulk sorbents and magnesium sulphate to remove residual water. Following clean-up, a small amount of formic acid was added to the extracts to improve the storage stability of certain base-sensitive pesticides. The obtained extracts were further analyzed using liquid chromatography with tandem mass spectrometry detection (LC-MS/MS) and gas chromatography with tandem mass spectrometry detection (GC-MS/MS). The procedure was conducted according to EN-15662:2008 “Foods of plant origin—Determination of pesticide residues using GC-MS and/or LC-MS/MS following acetonitrile extraction/partitioning and clean-up by dispersive SPE—QuEChERS-method” [60]. Quantification was carried out using an internal standard, which was added to the extracts after the initial acetonitrile addition. The method was calibrated for the concentration range 0.005–0.2 µg/mL (0.005–0.2 mg/kg in the sample). The samples containing pesticides in higher concentrations than 0.2 mg/kg (which equated to 0.4 mg/kg in dry and rehydrated samples) required additional dilution. For all measured compounds, the reporting limit was 0.01 mg/kg. Selectivity was guaranteed by the use of specific MRM transitions. The presence of each pesticide was proved by monitoring two MRM transitions originating from the decay of the parent ion into two different fragment ions. Linearity was evaluated based on calibration curves prepared using standard solutions and the determination coefficient. Some pesticides required the use of the quadratic regression model. Selected calibration curves are presented in the Supplementary Materials Figure S1.

## 5. Conclusions

The present study added significantly to broadening the knowledge of HBPs’ pesticide contamination in Europe. There is scarce information in the literature on the pesticide contamination of propolis and royal jelly. Moreover, to the best of our knowledge, this is the first report presenting the determination of pesticide residues in propolis and royal jelly of Polish origin. This current study revealed the high need for pesticide monitoring of HBPs and proved that there is a need to expand the European Union Pesticides Database and include more HBPs. A large number of pesticides, including insecticides, fungicides, and herbicides, were detected and quantified in propolis and pollen samples apart from different months and years of bee product collection. Therefore, the results indicated that bees in west-central Poland were exposed to multiple pesticides. Additionally, a comparison of pesticide contamination of HBPs from different parts of the world was discussed. The main limitation of this study is that only one apiary was involved. However, the analysis of different HBPs collected from the same area allowed us to compare the levels of pesticide residues between them and evaluate their role as potential bioindicators. We do not have data about the specific date of spraying and use of the pesticides, so there was no possibility of controlling the environmental contamination. Nevertheless, this study revealed that pesticides might penetrate into HBPs and thus have an influence not only on bees but also people who are consumers. Bearing in mind that the combination of different pesticides may increase their toxicity due to interactions, all detected pesticides should be considered as a potential danger and should be constantly monitored. The present publication indicates in the Discussion section that even low-toxicity pesticides may be hazardous depending on the duration and degree of total exposure. Therefore, future studies should investigate the long-term effects of low-level pesticide exposure on honeybee health. Furthermore, we also discussed the synergistic effects of exposure to multiple pesticides, and the exploration of these mechanisms may be a valuable area of further research.

## Figures and Tables

**Table 1:** Pesticide residue content for substances above LOQ measured using GC-MS/MS.

Pesticide Contamination	Class	Pollen August 2017	Pollen May 2018	Pollen June 2018	Pollen July 2018	Pollen May 2019	Pollen June 2019	Pollen July 2019	Propolis June 2018	Propolis June 2019	Royal Jelly June 2019
Anthraquinone [mg/kg]	insecticide	*	0.013	*	*	*	*	*	*	*	*
Biphenyl [mg/kg]	fungicide	*	*	*	*	0.011	*	*	0.039	0.042	*
Boscalid [mg/kg]	fungicide	0.15	*	*	*	*	*	*	*	*	*
Chlorpyrifos [mg/kg]	insecticide	0.4	0.02	0.14	0.16	0.014	0.091	*	0.11	0.088	*
Cypermethrin (sum of isomers) [mg/kg]	insecticide	0.08	*	*	*	*	*	*	*	*	*
Cyprodinil [mg/kg]	fungicide	*	0.094	*	*	*	*	*	*	*	*
Difenoconazole [mg/kg]	fungicide	*	*	0.015	*	*	*	*	*	*	*
Fenpropimorph [mg/kg]	fungicide	*	*	*	*	*	0.013	*	*	*	*
Fenvalerate (sum of isomers) [mg/kg]	insecticide	0.058	*	0.01	*	*	*	*	*	*	*
Fludioxonil [mg/kg]	fungicide	*	0.048	*	*	*	*	*	*	*	*
Pendimethalin [mg/kg]	herbicide	*	*	*	0.021	0.021	0.15	*	*	*	*
Propiconazole [mg/kg]	fungicide	*	*	*	*	*	*	0.037	*	*	*
Pyrimethanil [mg/kg]	fungicide	*	0.042	*	*	*	*	*	*	*	*
Tebuconazole [mg/kg]	fungicide	*	0.2	*	*	*	*	*	*	*	*

* Pesticide residue content below LOQ (0.01 mg/kg).

**Table 2:** Pesticide residue content for substances above LOQ measured using LC-MS/MS.

Pesticide Contamination	Class	Pollen August 2017	Pollen May 2018	Pollen June 2018	Pollen July 2018	Pollen May 2019	Pollen June 2019	Pollen July 2019	Propolis June 2018	Propolis June 2019	Royal Jelly June 2019
Acetamiprid [mg/kg]	insecticide	0.015	0.021	*	*	0.014	0.012	*	*	*	*
Azoxystrobin [mg/kg]	fungicide	2.9	0.57	*	0.02	*	0.018	0.017	*	*	*
Carbendazim [mg/kg]	fungicide	*	*	*	*	*	0.062	*	*	*	*
Chlorotoluron [mg/kg]	herbicide	*	0.011	*	*	*	*	*	*	*	*
Dimethoate [mg/kg]	insecticide	*	*	*	*	*	*	*	0.031	*	*
Diuron [mg/kg]	herbicide	*	0.029	*	*	*	*	*	*	*	*
Dodine [mg/kg]	fungicide	*	0.024	*	*	*	*	*	*	*	*
Flonicamid (Parent only) [mg/kg]	insecticide	*	0.014	*	*	*	*	*	*	*	*
Metamitron [mg/kg]	herbicide	*	0.014	*	*	*	*	*	*	*	*
Propamocarb Hydrochloride [mg/kg]	fungicide	*	*	0.18	*	*	*	*	*	*	*
Prosulfocarb [mg/kg]	herbicide	*	*	*	*	0.01	0.012	*	*	*	*
Pyraclostrobin [mg/kg]	fungicide	0.021	*	*	*	*	*	*	*	*	*
tau-Fluvalinate [mg/kg]	insecticide	*	*	*	*	*	*	*	0.05	*	*
Thiacloprid [mg/kg]	insecticide	*	*	0.015	*	*	*	0.18	*	*	*
Thiophanate-Methyl [mg/kg]	fungicide	*	0.016	*	*	*	0.043	*	*	*	*

* Pesticide residue content below LOQ (0.01 mg/kg).

**Table 3:** Pesticide residue content of pollen samples (*n* = 7) for substances with concentrations higher than maximum residue levels (MRLs) for honey.

Pesticide	Class	MRL for Honey [mg/kg]	Positive Casses	Range [mg/kg]	Mean [mg/kg]
Biphenyl	fungicide	0.01	1 (14.2%)	0.011	0.011
Boscalid	fungicide	0.05	1 (14.2%)	0.150	0.150
Chlorpyrifos	insecticide	0.01	6 (85.7%)	0.014–0.400	0.138
Cypermethrin (sum of isomers)	insecticide	0.05	1 (14.2%)	0.080	0.080
Cyprodinil	fungicide	0.05	1 (14.2%)	0.094	0.094
Fenvalerate (sum of isomers)	insecticide	0.05	1 (14.2%)	0.058	0.058
Tebuconazole	fungicide	0.05	1 (14.2%)	0.200	0.200
Azoxystrobin	fungicide	0.05	2 (28.6%)	2.900–0.570	1.735
Propamocarb Hydrochloride	fungicide	0.05	1 (14.2%)	0.180	0.180

## Data Availability

All data that support the findings of this study are included within this article.

## Supplementary Materials

The following supporting information can be downloaded at https://www.mdpi.com/article/10.3390/molecules30020275/s1. Figure S1: Calibration curves for the following pesticides: boscalid, propamocarb, azoxystrobin, tebuconazole, cyprodinil, cypermethrin, and chlorpyrifos.
