# Supplementary material for: Pesticides in Honeybee Products—Determination of Pesticides in Bee Pollen, Propolis, and Royal Jelly from Polish Apiary
Source: Molecules. 2025 Jan 12;30(2):275. doi: 10.3390/molecules30020275 (PMC11767846; doi:10.3390/molecules30020275)

ID351: Chlorpyrifos

Curve Fit: Linear | Weighting: None | Zero: Force Through

Quantitative Method: External Standard

Q 350.00>197.95

$R^2 = 0.9993246$ $R = 0.9996622$

$y = 19943270x + 0.0000000$

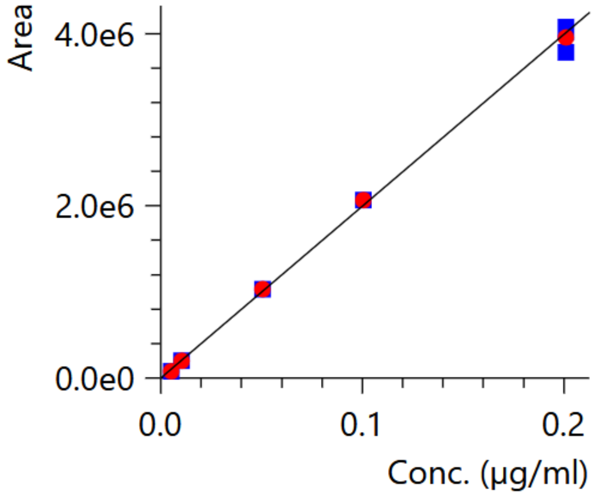

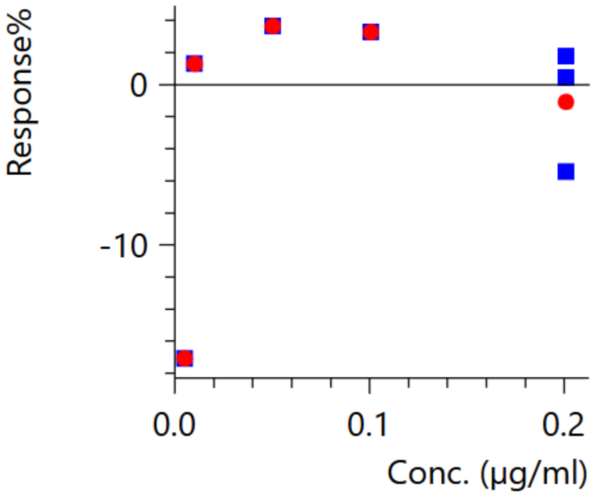

ID371: Cypermethrin

Curve Fit: Linear | Weighting: None | Zero: Force Through

Quantitative Method: External Standard

Q 433.10>191.00

$R^2 = 0.9984595$ $R = 0.9992294$

$y = 1377868x + 0.0000000$

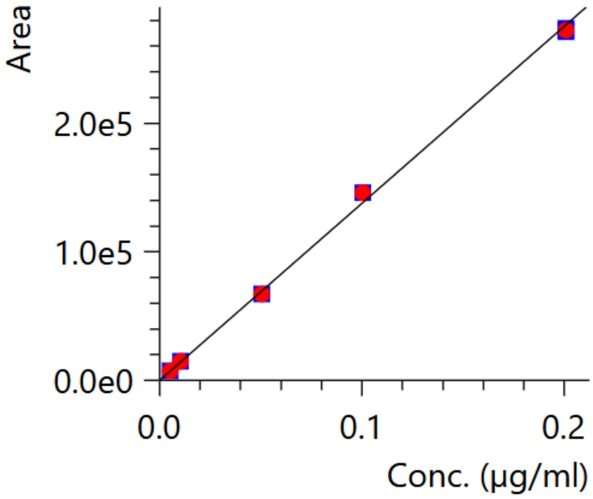

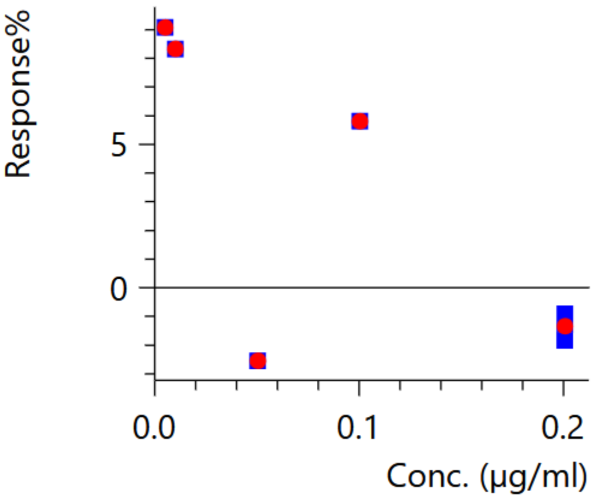

ID250: Cyprodinil

Curve Fit: Linear | Weighting: None | Zero: Force Through
Quantitative Method: External Standard
Q 226.10>93.00
R^2 = 0.9997539 R = 0.9998769
y = 10323560x + 0.0000000

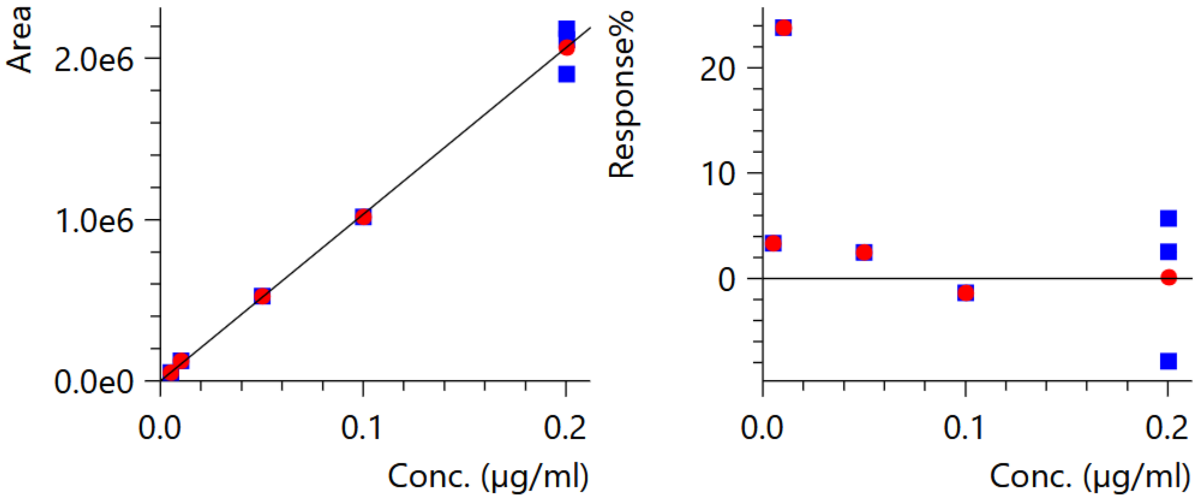

ID263: Tebuconazole

Curve Fit: Quadratic | Weighting: None | Zero: Force Through
Quantitative Method: External Standard
Q 308.20>70.05
R^2 = 0.9995953 R = 0.9997976
$y = -15391200x^2 + 28994450x + 0.0000000$

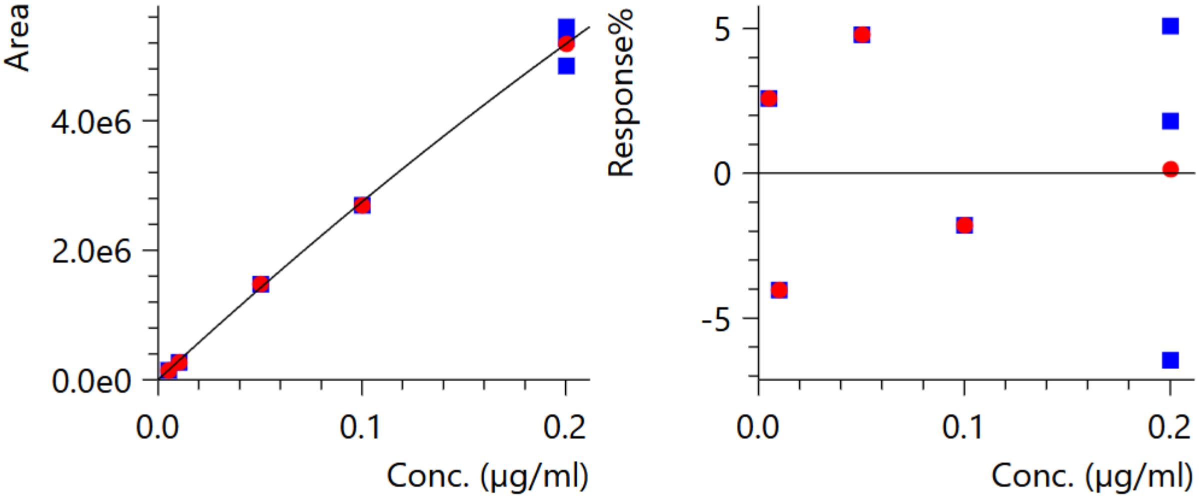

ID177: Azoxystrobin

Curve Fit: Linear | Weighting: None | Zero: Force Through

Quantitative Method: External Standard

Q 404.00>328.95

R^2 = 0.9995682 R = 0.9997841

y = 52945470x + 0.0000000

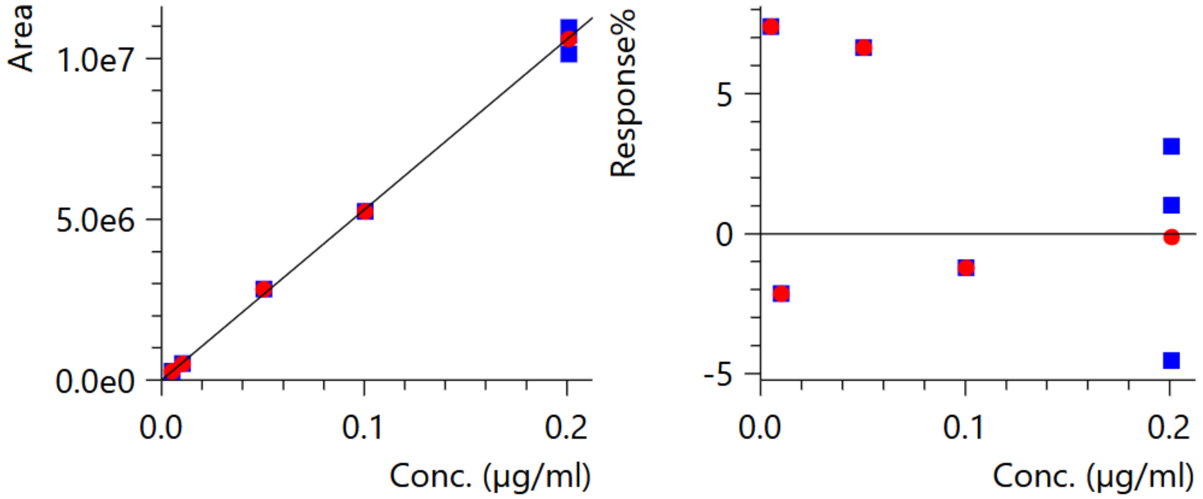

ID2: Propamocarb

Curve Fit: Linear | Weighting: None | Zero: Force Through

Quantitative Method: External Standard

Q 189.20>102.15

R^2 = 0.9996287 R = 0.9998143

y = 51063830x + 0.0000000

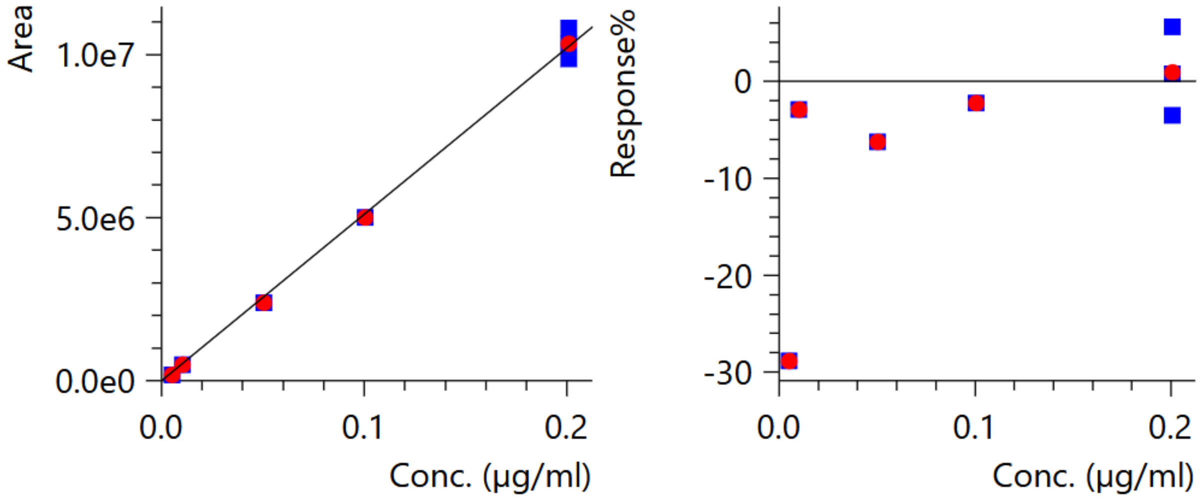

ID185: Boscalid

Curve Fit: Quadratic | Weighting: None | Zero: Not Forced

Quantitative Method: External Standard

Q 343.00>306.95

R^2 = 0.9999751 R = 0.9999875

$y = -5430505x^2 + 16538700x + 21472.12$

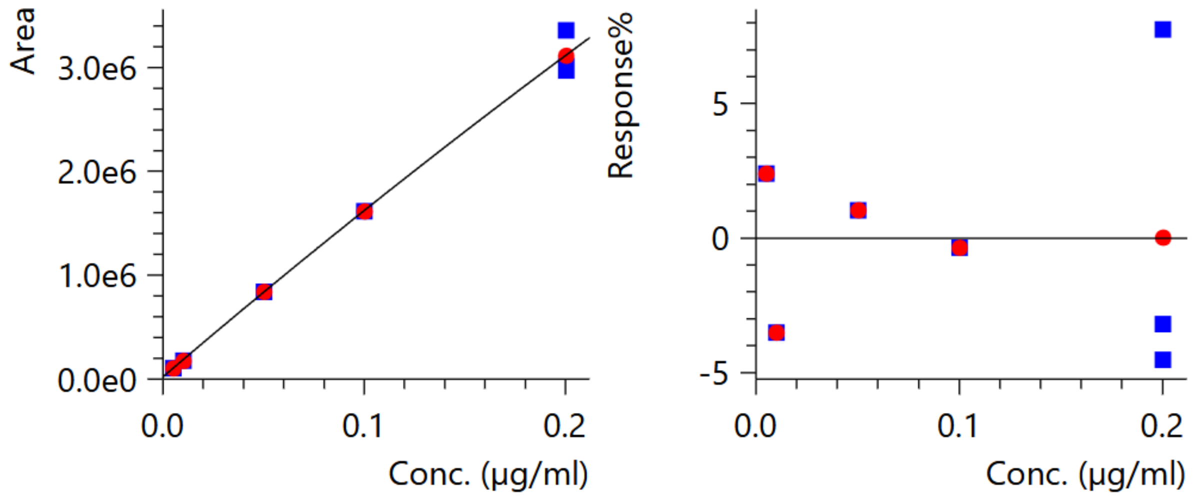

Supplement: Supplementary file 1 [file molecules-30-00275-s001.zip › molecules-3385251-supplementary.pdf]
